# A system for transferring large genetic loci in *Bacteroides* enables hemicellulose utilization in *Bacteroides thetaiotaomicron* and characterization of a locus from an uncultivated strain

**DOI:** 10.1128/aem.00176-26

**Published:** 2026-04-20

**Authors:** Nathan T. Porter, Cathleen Kmezik, Yi-Hsuan Lee, Verena Siewers, Phillip B. Pope, Nicole Koropatkin, Eric Martens, Johan Larsbrink

**Affiliations:** 1 Department of Life Sciences, Chalmers University of Technology 11248, Gothenburg, Sweden; 2 Faculty of Biosciences, Norwegian University of Life Sciences 56625, Ås, Norway; 3 Faculty of Chemistry, Biotechnology and Food Sciences, Norwegian University of Life Sciences 56625, Ås, Norway; 4 Centre for Microbiome Research, School of Biomedical Sciences, Queensland University of Technology, Translational Research Institute 568690, Woolloongabba, Australia; 5 Department of Microbiology and Immunology, University of Michigan Medical School 12266 , Ann Arbor, Michigan, USA; Kyoto University, Kyoto, Japan

**Keywords:** dietary fiber, gut microbiota, polysaccharide utilization loci, *Bacteroides*

## Abstract

Bacteria from the Bacteroidota phylum are well-known for their exceptional carbohydrate degradation abilities, which are closely associated with their use of polysaccharide utilization loci (PULs). The native exchange of PULs between different Bacteroidota species via horizontal gene transfer has been confirmed experimentally, and imitating such transfers *in vitro* can enable detailed studies of PULs from unstudied species in readily tractable hosts and the creation of novel strains. Here, we present a new set of tools and workflow to transfer large genetic loci, such as PULs, into genetically tractable *Bacteroides* strains. Our method uses a newly designed pICKUP plasmid and derivatives that allow site-specific genome integration and subsequent removal of the plasmid backbone. Thus, an unlimited number of loci may be transferred using the same resistance markers. We validated the pICKUP-based workflow by transfer of a previously characterized mixed-linkage *β*-glucan utilization locus (MLGUL) into the genome of *Bacteroides thetaiotaomicron,* which enabled metabolism of mixed-linkage *β*-glucan by this strain. Subsequent transfer of a PUL from an uncultivated bacterium from the bovine rumen confers the ability to metabolize certain cellooligosaccharides and allows further scrutiny of various genes in the PUL and their involvement in cellooligosaccharide metabolism. The pICKUP-based method presented here expands the toolkit for investigation of PULs and other large loci, including those from intractable or uncharacterized members of the Bacteroidota. A robust method to transfer whole PULs between species also forms the basis for creating custom-tailored PULs and resulting strains, with potential applications in improved gut health and biomass valorization processes.

**IMPORTANCE:**

The gut environment is a highly competitive niche, where dietary fiber is a major source of carbon, and polysaccharide utilization loci (PULs) encoded by Bacteroidota species are linked to their dominance in this environment. Our proposed method to study such PULs, especially from non-cultivated or unculturable strains, by transferring them into genetically tractable hosts, represents a valuable alternative to typical studies of isolated PUL-derived proteins. By transferring PULs into culturable hosts, we enable both a better understanding of the PULs’ biological roles and modification of the individual encoded genes. Our results show how a single PUL transfer can confer hemicellulose-degrading ability to *Bacteroides thetaiotaomicron*, which is lacking in the wild-type strain. Transfer of a PUL from a distantly related strain, speculated to confer cellulose-degrading capacity, showcases that this annotation may be incorrect and indicates that the utilization of foreign genetic material depends on species relatedness in the same phylum.

## INTRODUCTION

Bacteroidota species can be found in all environments, from guts to soils and aquatic ecosystems, and its members are renowned for being highly competitive degraders of complex polysaccharides of various origins (1–3). The efficient utilization of complex carbohydrates within the Bacteroidota is generally ascribed to their use of polysaccharide utilization loci (PULs) (4–6). Canonical PULs in this phylum are comprised of co-localized and co-regulated gene clusters that encode the proteins necessary for the degradation of a specific polysaccharide, and thus also confer the ability to grow on the target glycan.

The first PUL described was the starch utilization system (Sus) of the model gut bacterium *Bacteroides thetaiotaomicron* (7), which now serves as a blueprint for other PULs within the phylum. The details of the *B. thetaiotaomicron* Sus have been summarized in several excellent reviews (e.g., reference 4). Briefly, the Sus encodes a TonB-dependent transporter (SusC) that transports products from the substrate (starch) from the extracellular environment into the periplasm. The SusD binding protein binds to starch, provides structural stability, and may even form a lid for SusC that opens during transport of the oligosaccharides (8). Downstream of the *susC/D* gene pair, a pair of genes (*susE/F*) encodes additional cell surface-tethered starch-binding proteins (9). After the cell has bound to starch, three glycoside hydrolases (GHs) degrade the polysaccharide into glucose monosaccharides. The extracellular amylase SusG (GH family 13; GH13) cleaves the polysaccharide into smaller fragments, to be imported through SusC. Periplasmic enzymes SusA/B then degrade the oligosaccharides to glucose for import into the cytosol through a non-Sus-encoded transporter.

Apart from proteins for sugar capture, transport, and hydrolysis, the Sus and other PULs encode sensors/regulators that upregulate the whole locus upon sensing a breakdown product of the targeted glycan (4, 10). In general, PULs are believed to be constitutively transcribed at low levels, and upon sensing of certain oligosaccharides, the whole PUL is upregulated (10, 11). PULs targeting polysaccharides other than starch also have a similar functional setup but encode different carbohydrate-active enzymes (CAZymes) appropriate for the target glycan. Normally, there is only limited hydrolysis at the cell surface, followed by complete degradation to monosaccharides within the periplasm, though examples of secreted soluble enzymes have been found, for instance, in environmental species targeting crystalline chitin (12). Proteins homologous to the SusC/D pair (SusC/D-like proteins) are now used as signature motifs to identify new PULs in the genomes of Bacteroidota species, as exemplified by the PULDB database (https://www.cazy.org/PULDB/ [13, 14]).

Two of the best-studied Bacteroidota members of the human gut microbiome with regard to their PUL repertoires are the prevalent gut symbionts *B. thetaiotaomicron* and *Bacteroides ovatus*. These species encode around 100 PULs each, many of which are highly similar between both species, and accordingly, they are able to metabolize the same glycans to a large extent (11). However, *B. thetaiotaomicron* has a preference for degrading some pectins and host-derived *O*-glycans, e.g., mucus-derived polysaccharides, but is unable to metabolize plant biomass-derived hemicelluloses. In contrast, hemicelluloses are efficiently consumed by *B. ovatus* (11, 15). The number of highly similar PULs in the genomes of these two species and their synteny imply either a common ancestor and/or that PULs can be passed on between Bacteroidota species via horizontal gene transfer. Similarly, others have noted that syntenic PULs, i.e., with a highly similar gene organization, can be found in both closely and distantly related species (11, 16–18).

Many Bacteroidota loci reside within mobile genetic elements, including advantageous loci coding for antibiotic resistance, nutrient import, capsular polysaccharide synthesis, and PULs (19, 20). Evidence is building that locus transfers have occurred between both closely and distantly related Bacteroidota species and have transpired both anciently and recently (within a human individual) and provide competitive advantages to the recipient strains (15, 21–23). Of note, while many loci still encode the machinery necessary for self-excision and mobilization, loci with intact transfer machinery can evidently facilitate transmission of loci that have lost these functions (15, 24). We and others have recently demonstrated transfer of PULs and other loci through mobilization of native elements between *Bacteroides* strains (20, 23). In both of these cases, only certain combinations of donor and recipient strains achieved effective locus transfer, and it is unclear what conditions or genetic factors determine or restrict successful transfer.

Besides providing knowledge on native transfer mechanisms, experimental transfer of large loci would allow the study of a PUL or other locus in a controlled environment, e.g., if the native strain is uncultivable or lacks a well-established genetic toolkit. An example that we have targeted here is a putative PUL identified in a metagenomics study, where two of the PUL enzymes showed activity on cellulose (25). However, regulatory or import functions have not been characterized, and what degradative capability the PUL confers has not been confirmed. Second, locus transfer enables rational strain design for academic or biotechnological use. For instance, a recent plasmid vector incorporates a partial PUL for inulin degradation as a selective marker in antibiotic-resistant *Bacteroides* strains (26). Others have transferred a PUL for porphyran degradation into various *Bacteroides* strains, allowing them to demonstrate porphyran supplementation-dependent establishment of the PUL recipient strain in the mouse gut (27). This method relies on the functions of a mobile genetic element from *Bacteroides*, NBU2, that integrates into the genome at specific *att* sites and “consumes” them in the process (28, 29). While a newly isolated strain of *Bacteroides stercoris* successfully took up truncations of the PUL element between ~20 and 60 kbp in length, transformation into the model *B. thetaiotaomicron* strain required pre-integration and expression of a second construct before even the smaller PUL fragments (~20–40 kbp) would integrate. In this situation, both common antibiotic markers and both NBU2 insertion sites are “used up,” prohibiting straightforward integration of any more loci within the strain.

Here, we present an alternative method to transfer large loci that removes plasmid backbones and antibiotic markers, allowing for theoretically innumerable transfers to a single strain. The developed pICKUP plasmid system allows for a flexible decision on the genomic integration site, and we confirm two such sites here. We first validate the successful transfer of a well-characterized PUL from *B. ovatus* to *B. thetaiotaomicron*, which enabled a new polysaccharide utilization phenotype for the recipient, after which we employ our new method to characterize a PUL from an uncultured Bacteroidota strain from the bovine rumen. We confirm the role of this PUL in enabling cellooligosaccharide catabolism. Additionally, transfer of this PUL to a genetically tractable, well-studied organism enabled us to genetically characterize various PUL genes in a way currently impossible to do in the donor strain. This new method adds to the expanding genetic toolbox for *Bacteroides* species and enables straightforward study of PULs and other loci through import to model *Bacteroides* species, such as *B. thetaiotaomicron*.

## RESULTS

### Creation of the pICKUP plasmid

Recent efforts have led to a genetic system capable of integrating a PUL of impressive size (up to ~60 kbp) into two members of the *Bacteroides* (27). This integration method is based on NBU2 site-specific integration into the 3′ end of a serine tRNA sequence (28). While two smaller versions of this same PUL (~20–40 kbp) could integrate into the extensively studied model organism *B. thetaiotaomicron*, this occurred only after first integrating a second plasmid construct to upregulate expression of the NBU2 integrase, unfortunately, using both common *Bacteroides* antibiotic resistance markers. For our work, we desired a simpler plasmid system for *B. thetaiotaomicron* and its relatives that allowed greater flexibility in choosing the locus integration site, while also removing the vector backbone and antibiotic resistance genes for subsequent modifications. We chose to adapt the common suicide vector pExchange-*tdk* for these purposes (30). pExchange-*tdk* relies on integration through homologous “targeting region” sequences that flank the user-chosen integration site. Counterselection to remove the plasmid backbone occurs in specially generated strains missing thymidine kinase (encoded by BT2275, *tdk*) and results in a “clean” removal of the plasmid and a strain that either contains the plasmid insert or has reverted to the parental sequence. As *tdk* mutants already exist for the type strains of model organisms *B. thetaiotaomicron* and *B. ovatus*, and this mutation is readily generated for other strains (e.g., reference 27), a pExchange-*tdk*-based system should also be readily employable in a range of other strains and species and can enable studies of PULs otherwise uneasily testable in their native strains.

**Fig 1:**
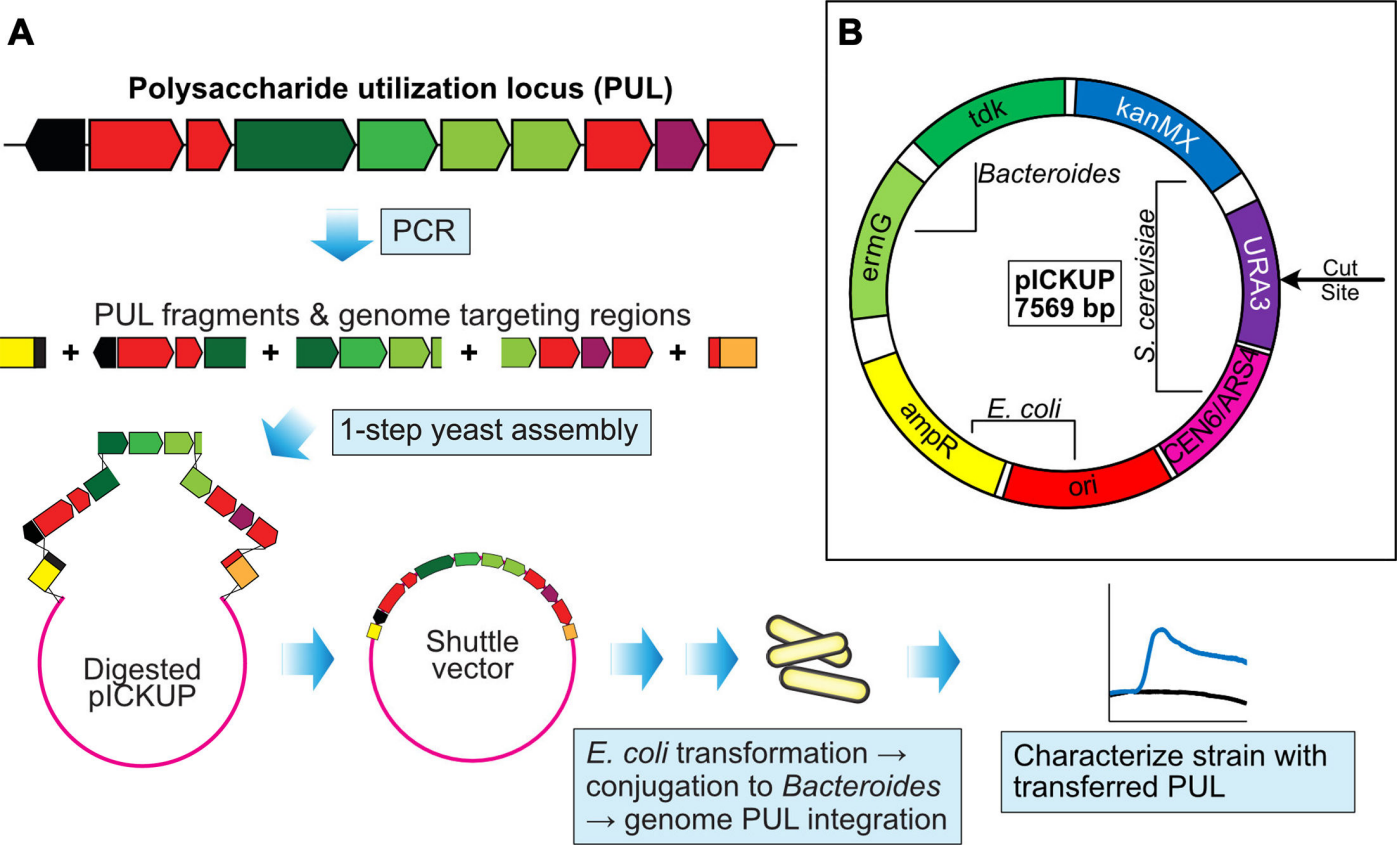
Overview of the pICKUP-based workflow for insertion of foreign PULs into *Bacteroides*. (**A**) After selection of a PUL of interest, overlapping fragments of the PUL are amplified by PCR. DNA flanking the chosen insertion site (targeting/homology regions) is also amplified with engineered overlaps to the PUL and to the pICKUP vector backbone. The various parts are assembled into a single vector by homologous recombination in *Saccharomyces cerevisiae*, before transferring the plasmid to *E. coli* and finally to the bacterial strain of interest. (**B**) Vector map of pICKUP, which contains factors for replication and selection in both *S. cerevisiae* and *E. coli*, as well as genes for selection and counterselection in *Bacteroides*.

We adapted pExchange-*tdk* into a new plasmid for PUL transfer by inserting a compact cassette encoding functions for yeast plasmid replication, antibiotic resistance, and uncut backbone counterselection into the multiple cloning site of pExchange-*tdk*. The derivative plasmid (pICKUP) is thus capable of receiving its large DNA cargo through yeast-mediated homologous recombination of the cut backbone with PCR-amplified fragments of the PUL or another locus of interest. Flanking targeting regions are amplified with overlaps to both the plasmid backbone and to the ends of the PUL (Fig. 1). Plasmid DNA is crudely extracted from yeast and transformed into *Escherichia coli*, prior to conjugation of this construct to the *Bacteroides* recipient.

### Identification of insertion sites

The pICKUP method allows for site-directed integration into the genome of the receptor strain (here *B. thetaiotaomicron* VPI-5482 *tdk*), by incorporating target sites homologous to a pre-selected genomic region in the receptor strain. It is not known if DNA transferred via bacterial conjugation can be integrated anywhere into the recipient genome or if the choice of insertion site could impair the integration process. In any case, integration that disrupts existing genes or other important genomic motifs should be avoided. To identify potential target sites, we performed a comparative analysis of published genome sequences of different *B. thetaiotaomicron* strains to identify genomic sites in which gene integration has naturally occurred, as was described previously for the identification of capsular polysaccharide synthesis locus insertions (31) (see Materials and Methods and Fig. S1). Two initial insertion sites for PUL insertion were chosen adjacent to BT0638 and BT2943 (Table 1) and named after the closest gene upstream of the site of integration (target region [TR] 0638 and TR2943, respectively). These and two other sites, TR0759 and TR2007, were briefly evaluated by inserting pICKUP with a small cargo (total ~12 kbp insertion), and integration was similarly successful in all sites, as determined by colony counts and PCR (data not shown).

**TABLE 1:** Overview of tested and successful insertion sites in *B. thetaiotaomicron tdk^a^*

Name	Location in genome	Insertion in reference genome
TR0638	Between BT0638 and BT0639	CRISPR locus
TR0759	Between BT0759 and BT0760	CPS synthesis operon
TR2007	Between BT2007 and BT2008	CPS synthesis operon
TR2943	Between BT2943 and BT2944	Other mobile genetic element

^*a*^ CRISPR, clustered regularly interspaced short palindromic repeats; CPS, capsular polysaccharide.

### Transfer of the *B. ovatus* MLGUL into *B. thetaiotaomicron*

While *B. thetaiotaomicron* is largely deficient in utilizing hemicelluloses, *B. ovatus* is able to metabolize all major plant hemicelluloses, including mixed-linkage *β*-1,3/1,4-glucan (MLG), which is a major polysaccharide in cereals (11). The MLG utilization locus (MLGUL) from *B. ovatus* confers the ability to degrade MLG and has been well-characterized (18, 32). Whereas the PULDB defines the MLGUL as spanning the genes BACOVA_02738-47 (13), compelling evidence has been presented to exclude BACOVA_02738 from the MLGUL (18). Here, we define the MLGUL of *B. ovatus* as spanning the genes BACOVA_02739 to BACOVA_02747.

**Fig 2:**
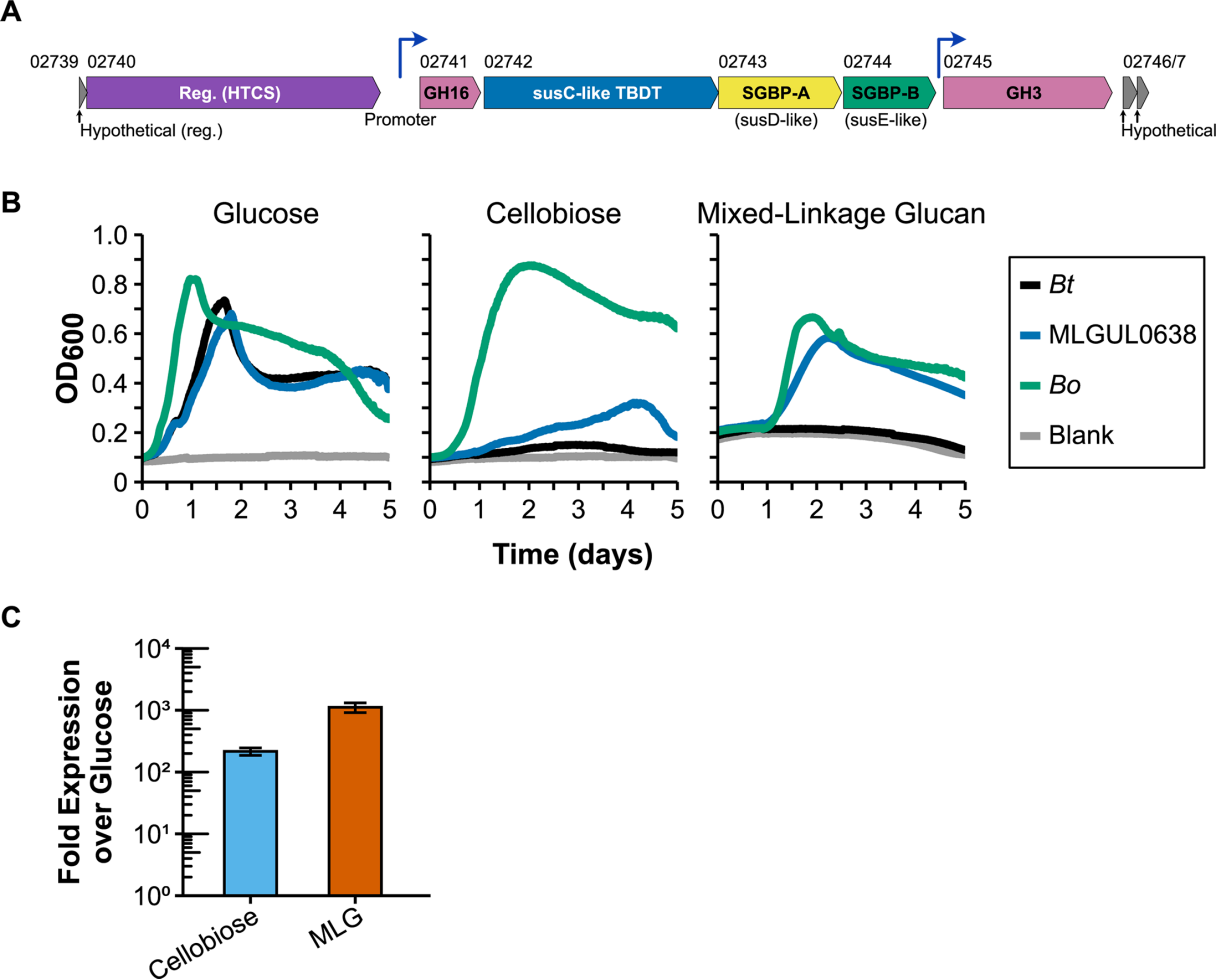
Transfer of the MLGUL from *B. ovatus* (*Bo*) to *B. thetaiotaomicron* (*Bt*). (**A**) Genetic overview of the MLGUL. Numbers above each gene correspond to locus tags (e.g., 02739 refers to BACOVA_02739). Blue arrows refer to promoter regions. (**B**) Growth of the donor, recipient, and transconjugant strains in minimal medium containing the indicated carbohydrates. (**C**) Expression of the MLGUL in Bt_MLGUL0638_ when grown to mid-log phase in minimal medium with the carbohydrates indicated. Reg., regulator; TBDT, TonB-dependent transporter; SGBP, surface glycan binding protein; MLG, mixed-linkage glucan.

Transfer of the relatively small MLGUL (16 kb) between these closely related species presented a suitable initial test of the pICKUP method (Fig. 2A). The MLGUL was PCR amplified in three pieces from 5 to 6 kbp in length, along with 1 kbp BT0638 targeting regions, each containing 40 bp overlaps to enable fusion of the MLGUL parts, the targeting regions, and the plasmid backbone. The insertion of cargo into the URA3 gene of the pICKUP backbone allows for selection against the uncut backbone, and the plasmid was assembled in yeast, transformed into *E. coli*, and inserted into the BT0638 insertion site as described in Materials and Methods. Proper integration of the MLGUL into *B. thetaiotaomicron*, yielding the new *Bt*_MLGUL0638_ strain, was validated by PCR screening, followed by Sanger sequencing. The *Bt*_MLGUL0638_ strain grew similarly to the wild-type parent strain (*B. thetaiotaomicron tdk*) in minimal medium with glucose, indicating insertion into the TR0638 site does not exert a noticeable detrimental effect on the cell (Fig. 2B).

Besides verifying insertion of the MLGUL, we confirmed MLG-induced expression of the locus in the *Bt*_MLGUL0638_ strain (Fig. 2C), and additionally compared the growth of *B. ovatus* (the donor), wild-type *B. thetaiotaomicron* (the recipient), and the *Bt*_MLGUL0638_ strain in minimal medium with glucose, cellobiose, or MLG as a carbon source. While all three strains efficiently metabolized glucose, *Bt*_MLGUL0638_ also exhibited weak growth on cellobiose, which induces MLGUL expression to a lesser degree than MLG (Fig. 2B and C). *B. ovatus* encodes several β-glucosidases and is able to metabolize various glycans containing β-linked glucosides (e.g., MLG, xyloglucan, glucomannan), which likely accounts for its superior growth on these substrates (Fig. 2B), and our results indicate that other, stronger inducers than cellobiose are required for proper induction of the MLGUL. While wild-type *B. thetaiotaomicron* could not metabolize MLG, the MLGUL recipient strain grew to similar optical densities on MLG as *B. ovatus,* when accounting for *B. ovatus* reaching a higher OD_600_ in all media conditions (Fig. 2B; Table S1). Expression of the MLGUL reached over 1,000-fold in *Bt*_MLGUL0638_, similar to expression in the donor (11). Thus, the transfer of the MLGUL successfully transferred the phenotype of MLG degradation to a similar level as in the donor, indicating a successful pICKUP-based workflow and that the PUL functioned effectively in the recipient host and gave a strong new phenotype.

### Transfer of a metagenomic-derived PUL into *B. thetaiotaomicron*

**Fig 3:**
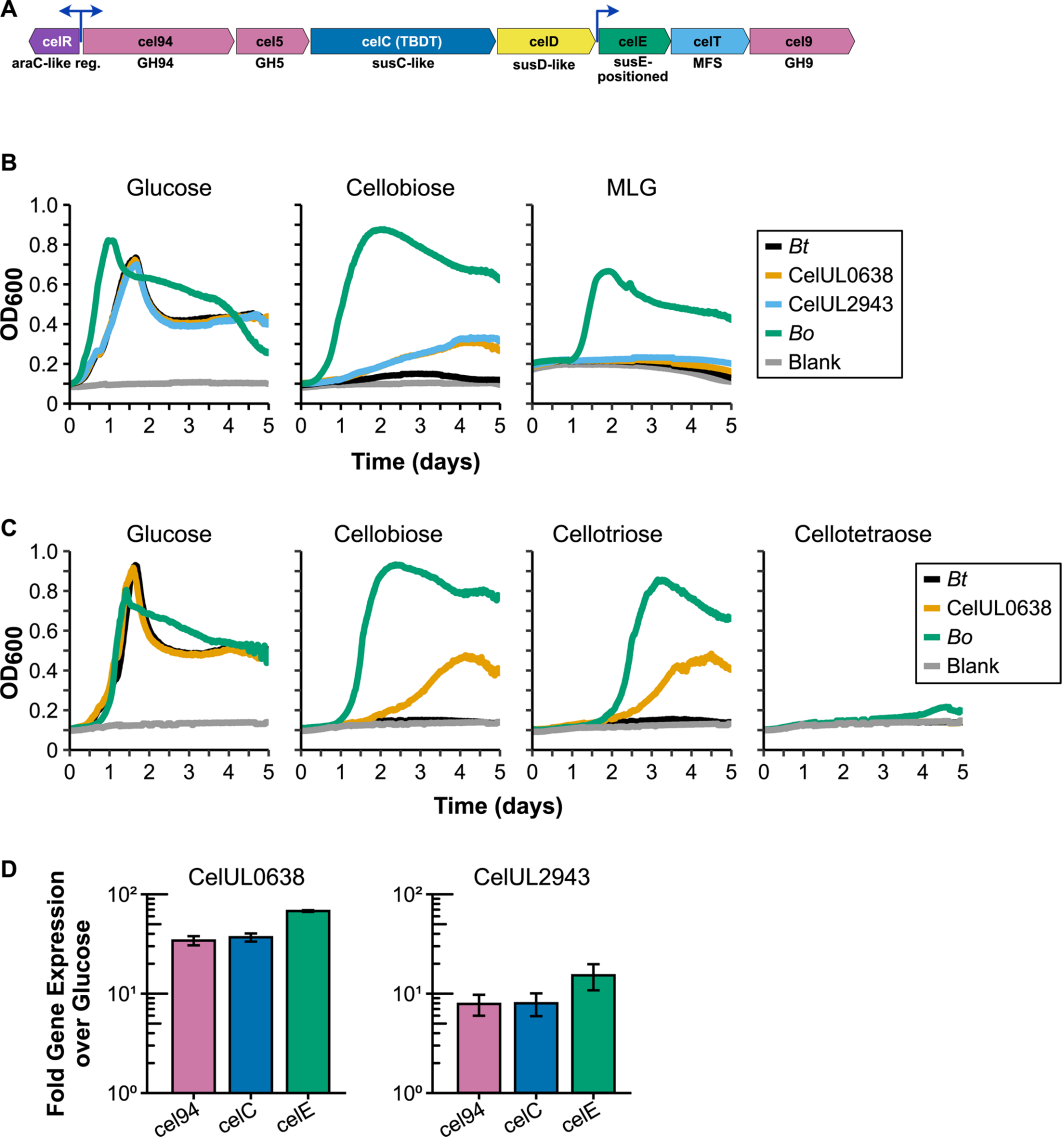
Transfer of the CelUL to *B. thetaiotaomicron* (*Bt*). (**A**) Genetic overview of the CelUL. Blue arrows refer to putative promoter regions. Descriptions underneath each gene indicate bioinformatic predictions of gene function. Reg., regulator; TBDT, TonB-dependent transporter; MFS, major facilitator superfamily transporter. (**B**) Growth of the donor, recipient, and the two transconjugant strains on minimal medium containing the indicated carbohydrates. MLG, mixed-linkage glucan. (**C**) Growth of the donor, recipient, and CelUL0638 transconjugant strains on glucose and cellooligosaccharides. (**D**) Change of expression of three CelUL genes in *Bt*_CelUL0638_ (left) and *Bt*_CelUL2943_ (right) in response to mid-log growth in minimal medium with cellobiose.

Encouraged by the successful MLGUL transfer, we chose to explore whether the pICKUP-based system could also transfer a PUL from a distantly related member of the Bacteroidota, leading to expression and enabling characterization. We selected a PUL previously identified in an uncultivated metagenomic sample. The predicted target polysaccharide of this PUL, hereafter termed “CelUL” (see Fig. 3A), is cellulose or related glucans, based on bioinformatic analyses and biochemical studies of some of its encoded enzymes. Cel5 and Cel9, from GH5 and GH9, respectively, were shown to be active on various glucans, such as carboxymethylcellulose, barley β-glucan, and lichenan, as well as filter paper (25, 33). The structure of Cel5 has been solved experimentally and was similar to characterized cellulases, xyloglucanases, and xylanases (33), and cleaved purified cellooligosaccharides DP4-DP6 into smaller fragments, whereas Cel9 cleaved DP3-6 to DP1-2 (25). Like the Sus, the CelUL encodes a SusC-like TonB-dependent transporter and SusD-like binding partner (termed CelC and CelD, respectively), as well as another “SusE-positioned” binding protein (CelE). Biochemical work focusing on CelD and CelE showed that each binds to barley β-glucan, filter paper, and partially to the highly crystalline cellulose Avicel (33).

Though targeting a glycan with only one constituent monosaccharide and two distinct linkages, the Sus encodes three GHs for starch degradation into glucose. Analogously, the CelUL encodes three GHs, including Cel5 and Cel9. The third enzyme, Cel94, has not been characterized but is predicted to be a cellobiose phosphorylase residing in the periplasm, cleaving cellobiose into glucose and glucose-1-phosphate, like other members of GH94. In contrast to the Sus, the CelUL also encodes a putative inner membrane transporter (CelT), which may enable transport of glucose-1-phosphate or other breakdown products to the cytosol. While the previous studies indicate a cellulolytic or β-glycan-targeting PUL, a more precise assessment is needed, and characterization of its function requires study of the intact PUL.

To study the CelUL in a recipient host, we first updated the pICKUP system by splitting the original vector into two separate vectors (pICKUP2A, pICKUP2B; Fig. S2). Each is required, along with an insert, to assemble the intact vector in yeast. Doing so greatly reduced background colony formation and allowed for quick integration of the CelUL into pICKUP2 (data not shown). The CelUL was transferred to two genomic sites (TR0638 and TR2943), and the first was genome-sequenced to confirm the correct insertion site and 100% identical sequence to the CelUL (see Materials and Methods).

We tested these two CelUL-encoding strains (termed *Bt*_CelUL0638_ and *Bt*_CelUL2943_) on an array of carbohydrate substrates, including those identified as substrates for the encoded GH5 and GH9 enzymes as described above. While displaying similar growth to wild-type *B. thetaiotaomicron* on glucose, receipt of the CelUL (shown in Fig. 3A) enabled weak growth on cellobiose but not on MLG (Fig. 3B; Table S1). Follow-up experiments revealed that the *Bt*_CelUL0638_ strain was unable to grow on any other polysaccharides tested, including carboxymethylcellulose, hydroxyethylcellulose, lichenan, cellulose (Avicel or milled filter paper), xyloglucan, or xylan (data not shown). We wondered whether the CelUL thus enabled growth on other cellooligosaccharides longer than cellobiose. Growth of the *Bt*_CelUL0638_ strain was also observed on cellotriose (C3), but not on cellotetraose (C4) or cellohexaose (C6; Fig. 3B; Table S2). Interestingly, the parental *B. thetaiotaomicron* sporadically exhibited a limited amount of growth on C3 (and sporadically minimal growth on C2) but not on the other cellooligosaccharides (Tables S1 and S2). It is possible that whatever native machinery is present for targeting C3 in *B. thetaiotaomicron* is augmented by one or more factors present in the CelUL. In comparison to the *B. thetaiotaomicron* strains, *B. ovatus* grows robustly on both cellobiose and cellotriose, likely thanks to its ability to metabolize multiple β-glucans.

The poor growth exhibited by the *Bt*_CelUL0638_ strain on even cellobiose led us to question whether the CelUL was highly expressed in medium with cellobiose. As compared to growth in minimal medium with glucose, cellobiose only induced the CelUL by ~30–70-fold in the *Bt*_CelUL0638_ strain and ~8–15-fold in the *Bt*_CelUL2943_ strain (Fig. 3C). In contrast, many PULs respond to their cognate substrate with 100–10,000-fold induction (16, 34). Thus, two or more factors could lead to low induction of the CelUL. First, the natural target for the CelUL may yet be unidentified. The MLGUL exhibits strong but lower induction during growth on cellobiose than on a more relevant substrate for the PUL: MLG (Fig. 2C [11]). Since *B. ovatus* targets many distinct glucans, it may be to its advantage to weakly induce several PULs in response to a metabolite shared by multiple polysaccharides of interest. In this situation, the cognate substrate for the CelUL would likely be similar in nature to cellobiose or contain cellobiose moieties in its structure. Second, the CelUL, though intact as witnessed by the genome sequence, may not be fully functional in *B. thetaiotaomicron*. This could be due to other, non-colocalized genes in the donor genome needed to support its growth on cellooligosaccharides or another substrate. Colocalization of all necessary genes in PULs is not as conserved in other members of the Bacteroidota like it is for gut-dwelling members (6). For instance, the marine bacteroidete *Zobellia galactanivorans* degrades carrageenan with multiple sets of PUL genes spread throughout the genome (35). Alternatively, regulatory sequences, such as the promoters from such a distant relative, may be inadequately recognized and upregulated in *B. thetaiotaomicron*. Further study of this PUL may lead to the identification of new substrates or a more clear understanding of genetic differences among members of the Bacteroidota.

### Genetic characterization points to the CelUL’s role in targeting cellooligosaccharides

While growth of the CelUL-containing strains was lackluster on cellooligosaccharides, transferring this PUL to a tractable relative with a large genetic toolkit allows us to characterize the PUL in ways not available previously. We focused on potential regulatory factors not easily investigated without the intact PUL, as well as on key genes that could be necessary for enabling C2 and C3 degradation in the *Bt*_CelUL0638_ strain.

**Fig 4:**
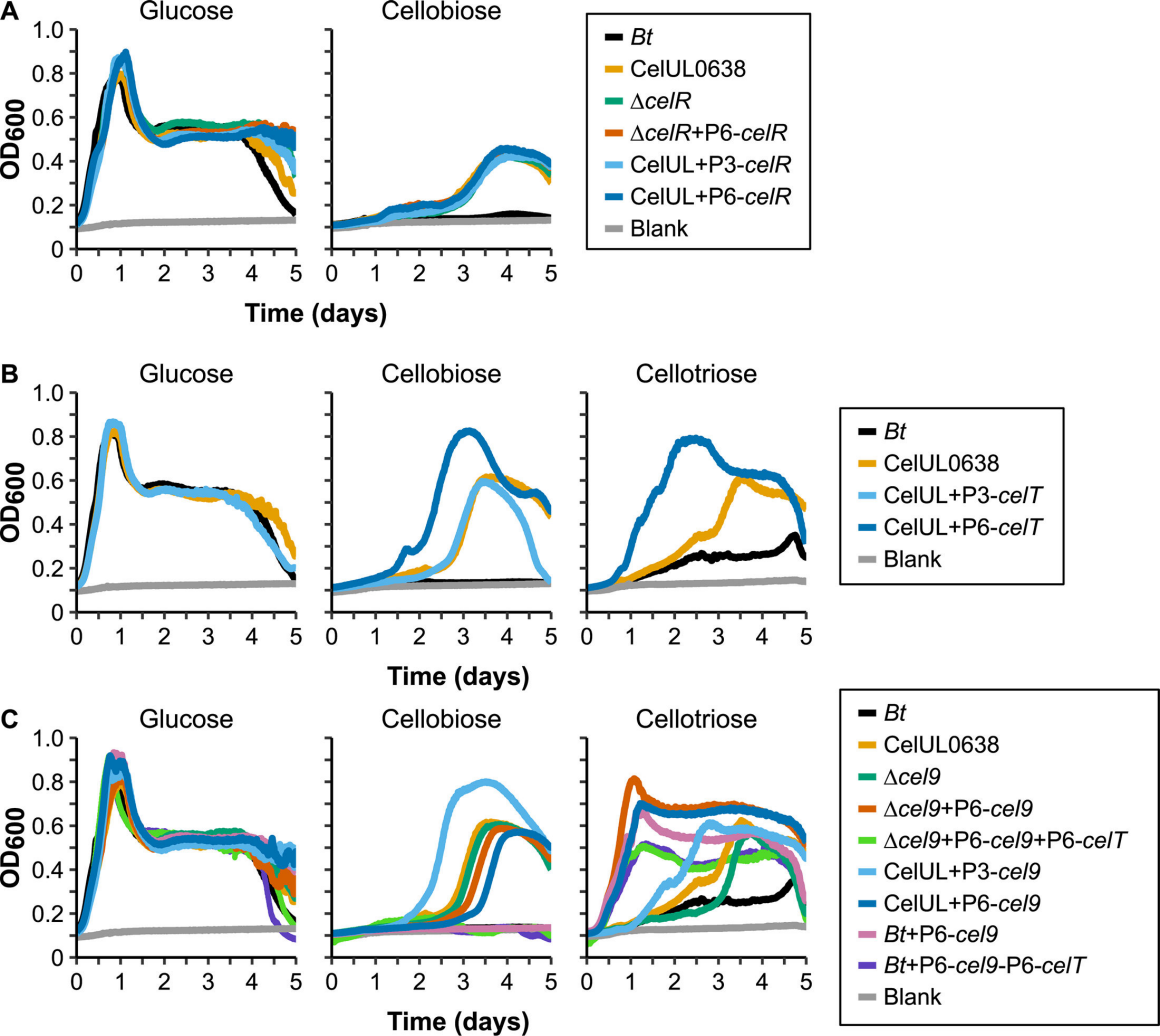
Growth characterization of strains with specific modifications to the CelUL. Growth in minimal media of wild-type *B. thetaiotaomicron* (*Bt*), *Bt*_CelUL0638_ strain (CelUL0638), and strains with various gene deletions or gene overexpressions. Other than *Bt+*P6-*cel9* and *Bt*+P6-*cel9*-P6-*celT* [the *cel9* and/or *cel9+celT* gene(s) overexpressed in the wild-type strain], all modifications occurred in the CelUL0638 strain. Strains were grown with the carbohydrate indicated above each panel. (**A**) *celR* modifications. (**B**) *celT* modifications. (**C**) *cel9* modifications. P3, promoter P_BgP2E3; P6, promoter P_BfP2E5.

First, the CelUL appears to encode up to three promoter sequences (denoted in Fig. 3A) based on gene direction, DNA folding (mfold [36]), and presence of canonical or near-canonical *Bacteroides* regulatory motifs (e.g., sequence motifs close to TATCTTTG or TTTG). Given poor upregulation of the CelUL in our experiments, we reasoned that we could increase expression of the PUL through the use of a constitutive promoter. Replacing the promoter sequence in front of *cel94* with a highly-expressed native *B. thetaiotaomicron* promoter (P_BT4615_) did not upregulate expression of the CelUL (data not shown) nor change growth parameters (Table S3). We also investigated the effect of the potential regulator (CelR) through deletion, complementation, and overexpression of this gene. However, none of these modifications altered growth on cellobiose (Fig. 4A; Table S4). Given its homology to other known regulators and its conserved position in the PUL, the lack of effect may be another indication that the native PUL sequence does not properly function in *B. thetaiotaomicron*.

Since the Sus depolymerizes all its target oligosaccharides into the common metabolite glucose, no special inner membrane transporter is needed. However, this is not the case for the CelUL. The PUL-encoded Cel94presumably acts on cellobiose or cellooligosaccharides to cleave off glucose-1-phosphate through an inverting mechanism. The CelUL-encoded putative inner membrane transporter (CelT) could thus be responsible for the transport of glucose-1-phosphate. Expression of *celT* under two different constitutive promoters showed divergent phenotypes: one promoter (P_BfP2E3) led to slightly reduced growth on cellobiose, whereas the other (P_BfP2E5, which enables higher expression [37]) consistently increased both growth rate and maximum growth (Fig. 4B; Table S4). Overexpression of *celT* with the P_BfP2E5 promoter also increased growth rate and max growth on cellotriose and led to short lag time on this oligosaccharide (Fig. 4B). This suggests that expression of this transporter is necessary or extremely beneficial for growth on these cellooligosaccharides in *B. thetaiotaomicron* and may also partly be the cause for the poor growth enabled by the native CelUL strain (*Bt*_CelUL0638_).

While the GH9 enzyme (Cel9) has been characterized *in vitro* (25), we next sought to determine its role in the whole PUL. As Cel9 degraded C3-6 to C1-2, the *cel9* gene should not be necessary for the metabolism of glucose or cellobiose, but Cel9 expression could be necessary or beneficial for growth on larger cellooligosaccharides. As expected, deletion of *cel9* did not affect growth on glucose or cellobiose (Fig. 4C; Table S4). However, *cel9* mutants exhibited a short lag while grown on cellotriose; thus, while the presence of Cel9 leads to quicker response to this cellooligosaccharide, its function in this case is redundant, possibly through the action of Cel5. Overexpressing the *cel9* gene with the P_BfP2E5 promoter in the *Bt*_CelUL0638_ strain or one with the *cel9* deletion led to higher levels of growth on cellotriose, and at a faster rate (Table S4). Interestingly, deletion of *cel9* with restoration of the *cel9 in trans* (removing the *cel9* gene from its normal regulatory context; “Δ*cel9*+P6-*cel9*”) allows for even faster growth on cellotriose. This fits the reasoning that the CelUL functions poorly due to low expression. In support of this, expressing only the *cel9* in the wild-type *B. thetaiotaomicron* strain (*“Bt+*P6-*cel9*,” Fig. 4C) leads to similar levels of growth on cellotriose, even without the presence of the CelUL. The wild-type strain is generally incapable of utilizing cellobiose, and growth must thus be dependent on the glucose liberated by Cel9 (Table S4), and the data support it being key in the degradation of cellotriose. Since *B. thetaiotaomicron* has two *att* sites capable of receiving pNBU2 vector insertion, we added P_BfP2E5-*celT* to the “*Bt+*P6-*cel9*” and “Δ*cel9*+P6-*cel9*” strains to create “*Bt+*P6-*cel9*+P6-*celT*” and “Δ*cel9*+P6-*cel9*+P6-*celT,*” following the same methods described in Materials and Methods. Despite the theoretical add-on effects, no enhanced phenotype was observed on cellooligosaccharides (Fig. 4C).

## DISCUSSION

Our work describes a straightforward method for transferring entire genetic loci into the well-characterized type strain of *B. thetaiotaomicron*. We identified multiple new insertion sites for these loci in the *B. thetaiotaomicron* genome and outlined a way to identify as many more as needed through comparative genomics. This, along with the fact that the antibiotic markers are removed during counterselection, allows for repeated insertion of genes, cassettes, or even larger loci into the genome. It is worth pointing out that repeated transfers to a single recipient strain may lead to accumulation of spontaneous point mutations, rearrangements, or other undesirable effects that, for example, could be assessed using whole-genome sequencing or transcriptomics. Recent efforts have shown that *B. thetaiotaomicron* can be engineered to produce butyrate through reconstitution of a several-step heterologous pathway (38). Production of this or other therapeutic molecules in gut-dwelling members of the Bacteroidota has great potential for treatment of intestinal disease *in situ*. However, engineering butyrate production, as well as refinement of the strain to boost production, required integration of both common *Bacteroides* antibiotic markers. Our pICKUP system can facilitate the expansion of these efforts to introduce multiple pathways sometimes required to produce molecules of interest, without the permanent introduction of antibiotic resistance genes to the organism.

Our data from the transferred MLGUL illustrate the potential for a PUL to work as well in the recipient as in the donor. Encouraged by the success of the MLGUL insertion, we attempted a “moonshot” by transferring a PUL from a metagenomic sample. Similar to the MLGUL, the CelUL transferred readily to *B. thetaiotaomicron* and was upregulated in response to cellobiose, but to a low extent. The factors that influenced its poor function are unknown, though it may be due to an alternate preferred substrate, differences in regulatory factors between the strains, or the absence of other important factors encoded elsewhere in the donor genome. Regardless, while previous studies showed individual enzymes and binding proteins could act on a range of glucans and marginally on xylans, our work specifically points to the CelUL as a cellooligosaccharide-targeting PUL. Overexpression of the genes encoding the inner-membrane transporter (*celT*) or the GH9 enzyme (*cel9*) led to enhanced growth on the cellooligosaccharides cellobiose and cellotriose, while double enhancement (*celT*+*cel9*) did not lead to further improvement. Cellulose itself is largely inaccessible to many carbohydrate-acting enzymes, as the linear chains can tightly pack together and impede chain transport to the enzyme active site. Enzymes such as lytic polysaccharide monooxygenases that are known to target tightly packed cellulose surfaces have thus far not been found within the Bacteroidota (6). Thus, the CelUL donor organism could scavenge fragments liberated by other primary cellulose degraders. In the mammalian large intestine, primary degraders, such as *Ruminococcus bromii,* play a similar role in degrading resistant starch, and *B. thetaiotaomicron* can scavenge and utilize the sugar released from *R. bromii* for its own growth (39). Alternatively, the CelUL donor organism could use a separate mechanism for hydrolyzing cellulose, and then the CelUL could provide a role in degrading and importing the smaller fragments.

The CelUL has not been studied in its native host, which was detected in the rumen rather than the intestine (40). Furthermore, Blastp searches for multiple of the CelUL proteins to identify the closest homologs (88%–99% amino acid identity) indicated them mainly being found in *Paludibacteraceae* (a different family than the *Bacteroidaceae* in the Bacteroidota phylum) metagenome-assembled genomes from ruminant gastrointestinal samples (data not shown). Thus, it is not likely to undergo native transfer to *Bacteroides* species. Even though the CelUL appears to function poorly upon transfer, we showed that even such a distantly related PUL could be expressed and enable new functionality in *B. thetaiotaomicron*. Furthermore, we were able to increase the CelUL’s effectiveness with straightforward modifications, such as gene overexpression, using *Bacteroides*-optimized promoters. Thus, the repertoire of PULs available for transfer to *B. thetaiotaomicron* is quite large and likely extends far beyond the limits of the *Bacteroides* genus, potentially to the *Bacteroidales* order, the *Bacteroidia* class, or even the entire phylum. Future work will continue to test the limits of the pICKUP system, including the effects of PUL size and evolutionary distance. Hopefully, more novel PULs can be genetically characterized in this manner, providing a window into the workings of many species that are as of yet understudied.

## MATERIALS AND METHODS

### Strains and media

Strains and plasmids used in this study are listed in Table S5. Formulations of growth media used are listed in Table S6. *S. cerevisiae* was grown in YPD medium at 30 °C; geneticin (200 µg/mL) was used as appropriate. *E. coli* was grown in LB medium at 37 °C, with ampicillin (100 µg/mL) or chloramphenicol (25 µg/mL) as appropriate. *B. thetaiotaomicron* strains and *B. ovatus* were grown in *Bacteroides* minimal medium for growth assays, and in rich TYG medium otherwise. For genetic manipulation, strains were grown on blood agar (BA) plates with gentamicin (200 µg/mL), erythromycin (25 µg/mL), or floxuridine (200 µg/mL) as appropriate. Unless otherwise indicated, *Bacteroides* were grown anaerobically at 37 °C in a Coy anaerobic chamber using a gas mix of 10% H_2_, 10% CO_2_, and balance N_2_. Glucose was purchased from Thermo Scientific, cellobiose, cellotriose, cellotetraose, cellohexaose, barley β-glucan (mixed-linkage glucan; MLG), tamarind xyloglucan, birch xylan from Megazyme (now Neogen), carboxymethylcellulose, hydroxyethylcellulose, and Avicel from Merck, filter paper from Whatman, and lichenan (*Cetraria islanidica*) from Carbosynth.

### Creation of pICKUP-based plasmids

pICKUP was created through traditional restriction/ligation cloning of pExchange-tdk (30) with a cassette containing kanMX, URA3, and CEN6/ARS4. For pICKUP2 vectors, part of pICKUP was amplified through PCR and restricted/ligated with a GFP cassette flanked by Eco147I (StuI isoschizomer) restriction sites to create pICKUP2A. pICKUP2B was created through PCR and restriction/ligation of another fragment of pICKUP into pNTP201 (41). Fragments used in derivative plasmids of the pICKUP system (e.g., targeting sequences, PUL fragments) were amplified by PCR and assembled into Eco147I -digested pICKUP via yeast-mediated homologous recombination (below). Derivatives of pICKUP2 were similarly created except that pICKUP2A was digested with Eco147I, whereas the pICKUP fragment in pICKUP2B was amplified by PCR and included as another piece for assembly by homologous recombination. pICKUP and pICKUP2A/B and annotated sequence files can be found in the Addgene repository with deposit number 82766.

### Identification of potential insertion sites in *B. thetaiotaomicron*

Out of 16 available genomes of *B. thetaiotaomicron* on IMG at the time of analysis (https://www.img.jgi.doe.gov [42]), the genome assembly of *B. thetaiotaomicron* 7730 (IMG genome ID 2844364567) was chosen as the reference genome, because of its “finished” sequencing status and high gene count. The Genome Gene Best Homologs tool was used to compare each gene in the reference genome to those in other available genome assemblies, including the VPI-5482 strain modified in this work. This revealed several sites in which genes or loci have been introduced in 7330 and other *B. thetaiotaomicron* strains, but that are not present in the VPI-5482 genome. These sites were cross-checked using the Gene Ortholog Neighborhoods feature of IMG and through Blast searches to genes within the locus.

### Yeast homologous recombination and plasmid extraction

Yeast homologous recombination was used to assemble pICKUP-based plasmids with PCR-amplified DNA of the target regions and locus inserts. Primers are listed in Table S7. Frozen competent yeast cells were prepared and transformed using the lithium acetate/single-stranded carrier DNA/polyethylene glycol method as directed in reference 43. A 300–500 ng of the backbone vector was used, along with an approximately equimolar amount of all inserts. Cells underwent heat shock at 42 °C for 40 min before centrifugation and resuspension in 1 mL YPD. Then, cells were incubated for 2–3 h (30 °C) before plating 100 µL of varying dilutions on YPD + geneticin (G418) plates, followed by incubation for 2–4 days at 30 °C.

For crude plasmid extraction, *S. cerevisiae* cells were scraped off a plate, diluted, and grown overnight in 5 mL of YPD + geneticin (30 °C, 180 rpm). The plasmid was extracted as previously described using glass beads and phenol:chloroform (44), prior to concentration by isopropanol precipitation. The pellet was resuspended in 10–20 µL of sterile water, of which 1–2 µL was used for transformation into *E. coli* S17-1 λpir.

### Transformation into electrocompetent *E. coli*

Competent *E. coli* S17-1 λpir were produced by growing *E. coli* S17-1 λpir in SOB medium at 37 °C (180 rpm) until an OD_600_ of 0.4 was reached. The cells were harvested by centrifugation (3,000 × *g* for 10 min) and serially washed in 1.0 and 0.5 volumes of ice-cold sterile water and 0.1 volumes of 10% vol/vol glycerol. Cells were then resuspended in 0.008 volumes of 10% vol/vol glycerol, aliquoted, snap-frozen with liquid nitrogen, and stored at −80 °C until further use. Ice-thawed competent *E. coli* cells were electroporated with the plasmid in a MicroPulser (Bio-Rad; setting Ec 2 at 2,500 V). Cells recovered for 1 h at 37 °C (180 rpm) in 1 mL LB prior to plating on LB with appropriate antibiotics and incubated at 37 °C for 18–48 h.

### Genetic manipulation of *Bacteroides*

Insertion of genetic loci into *Bacteroides thetaiotaomicron* was performed in the same manner as for gene deletions, as previously described (30). Briefly, mid-log phase *E. coli S17-1* λpir with a plasmid of interest was twice washed in fresh medium before being resuspended with *B. thetaiotaomicron* in TYG medium and plated on blood agar plates without antibiotics. After 1 day of aerobic incubation at 37 °C, dilutions of the bacterial lawn were twice plated on blood agar plates with erythromycin and gentamicin and incubated under anaerobic conditions at 37 °C for 2–3 days each time. Resulting colonies were grown overnight in TYG medium before being twice plated on blood agar plates with floxuridine for 2–3 days each time. Final colonies were screened by PCR, Sanger sequencing, and growth assays to confirm insertion of PUL sequences. To verify correct insertion of the CelUL, genome sequencing of the *Bt*_CelUL0638_ strain was performed at SeqCenter (Pittsburgh, PA, USA) with sample libraries prepared using the Illumina DNA Prep kit and sequenced on an Illumina NextSeq 2000. Demultiplexed, trimmed reads from SeqCenter were assembled *de novo* using SPAdes 3.15.4 (45), and the PUL was verified to be inserted at the TR0638 genomic site with 100% nucleotide identity to the expected sequence.

### Growth assays

Overnight cultures of *Bacteroides* strains were washed twice in double-strength minimal medium (no carbon source) before being resuspended 1:50 in the same medium. A 100 µL of resuspended cells was added to 100 µL of 10 mg/mL carbon source in a 96-well plate, with 1–3 technical replicates per condition. Optical density at 600 nm (OD_600_) was measured every 10 min for up to 4 days of anaerobic growth in a Sunrise microplate reader (Tecan, Switzerland) with internal temperature set to 36 °C.

## SUPPLEMENTAL MATERIAL

Supplemental material aem.00176-26-s0001.pdf Fig. S1 and S2; Tables S1 to S7.

ASM does not own the copyrights to Supplemental Material that may be linked to, or accessed through, an article. The authors have granted ASM a non-exclusive, world-wide license to publish the Supplemental Material files. Please contact the corresponding author directly for reuse.
