# Supplementary material for: A system for transferring large genetic loci in Bacteroides enables hemicellulose utilization in Bacteroides thetaiotaomicron and characterization of a locus from an uncultivated strain
Source: Appl Environ Microbiol. 2026 Apr 20;92(5):e00176-26. doi: 10.1128/aem.00176-26 (PMC13188876; doi:10.1128/aem.00176-26)
Supplement: Supplemental material — Fig. S1 and S2; Tables S1 to S7. [file aem.00176-26-s0001.pdf]

Supplemental information for:

A system for transferring large genetic loci in *Bacteroides* enables hemicellulose-utilization in *Bacteroides thetaiotaomicron* and characterization of a locus from an uncultivated strain

Nathan T. Porter[1], Cathleen Kmezik[1], Yi-Hsuan Lee[1], Verena Siewers[1], Phil Pope[2,3,4], Nicole Koropatkin[5], Eric Martens[5], Johan Larsbrink[1]*

[1]Department of Life Sciences, Chalmers University of Technology, SE-412 96 Gothenburg, Sweden

[2]Faculty of Biosciences, Norwegian University of Life Sciences, 1432 Ås, Norway

[3]Faculty of Chemistry, Biotechnology and Food Sciences, Norwegian University of Life Sciences, 1432 Ås, Norway

[4]Centre for Microbiome Research, School of Biomedical Sciences, Queensland University of Technology, Translational Research Institute, Woolloongabba, Australia

[5]Department of Microbiology and Immunology, University of Michigan Medical School, Ann Arbor, MI, 48109, USA

* correspondence: johan.larsbrink@chalmers.se

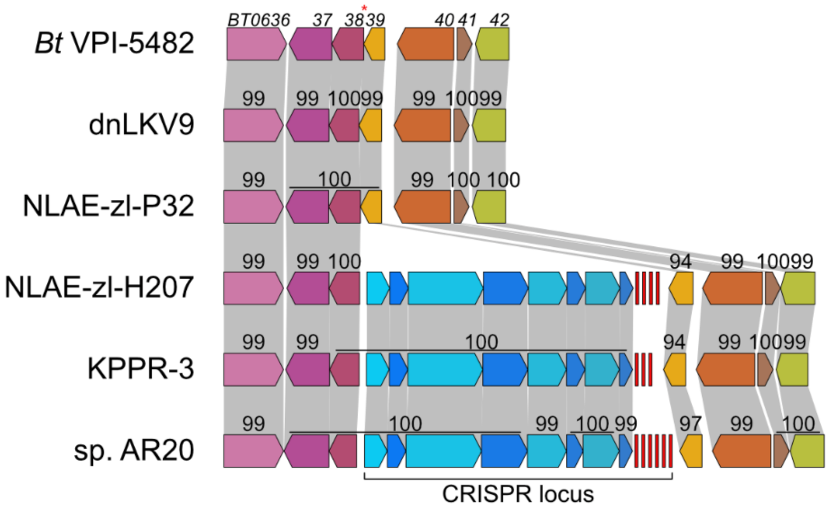

Figure S1. Comparison of the *B. thetaiotaomicron* genomic neighborhood near BT0638. Using the Integrated Microbial Genomes site (see *Methods*), the genomes of several *B. thetaiotaomicron* strains were compared to identify sites containing genetic loci in some strains but not in VPI-5482 (type strain used in this study). The BT0638 insertion site is shown here (marked with *), with numbers above genes in VPI-5482 referring to locus tags. Genes are color-coded by homology, and numbers above genes in other strains refer to percent amino acid identity to the corresponding VPI-5482 gene. Three of the strains shown encode a CRISPR locus at this site with no homology to the genes in VPI-5482; thus, numbers above CRISPR genes refer to percent amino acid identity to the CRISPR locus of NLAE-zl-H207.

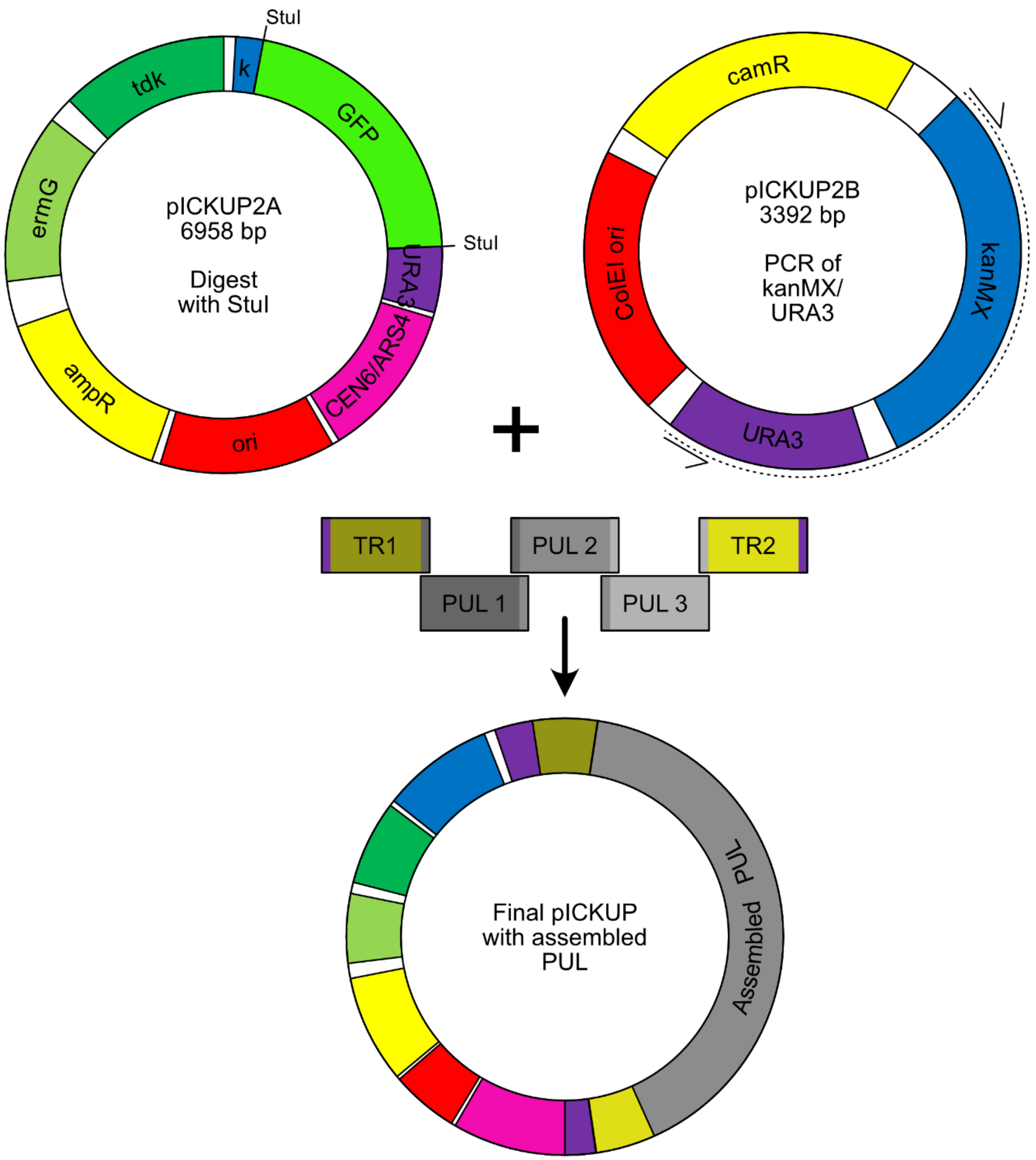

Figure S2. Splitting of the pICKUP vector. A fragment containing the majority of the *kanMX* and *URA3* genes (for selection and counterselection in *S. cerevisiae*) was replaced by GFP to create pICKUP2A. The missing fragment with additional sequence homologous to pICKUP2A was inserted into a separate vector, labeled pICKUP2B. Neither vector is capable of transforming yeast with high efficiency (data not shown), greatly reducing background with intact plasmid. Transforming StuI-cut pICKUP2A, PCR-amplified *kanMX/URA3* from pICKUP2B, and targeting (TR) and PUL sequences results in an assembled pICKUP vector with the same sequence as if using the original pICKUP.

Table S1. Bacterial growth characteristics on selected carbon sources (n = 3).

Carbon source	Strain	Total growth[a]	Specific growth rate (h^{-1})[b]	Lag Time (h)[c]
Glucose	*B. thetaiotaomicron*	0.73 ± 0.09	0.0225 ± 0.0043	12.92 ± 0.36
	CelUL0638	0.71 ± 0.08	0.0211 ± 0.0036	12.89 ± 0.27
	CelUL2943	0.70 ± 0.09	0.0206 ± 0.0045	12.50 ± 0.51
	MLGUL0638	0.71 ± 0.11	0.0200 ± 0.0047	12.61 ± 0.65
	B. ovatus	0.91 ± 0.10	0.0416 ± 0.0050	10.94 ± 1.88
Cellobiose	*B. thetaiotaomicron*	0.04 ± 0.01[d]	0.0007 ± 0.0002	N/A
	CelUL0638	0.27 ± 0.07	0.0032 ± 0.0008	48.64 ± 4.05
	CelUL2943	0.29 ± 0.05	0.0033 ± 0.0009	45.72 ± 5.08
	MLGUL0638	0.27 ± 0.05	0.0032 ± 0.0007	46.56 ± 5.69
	B. ovatus	0.80 ± 0.03	0.0196 ± 0.0008	17.58 ± 1.23
MLG	*B. thetaiotaomicron*	No growth	No growth	No growth
	CelUL0638	No growth	No growth	No growth
	CelUL2943	No growth	No growth	No growth
	MLGUL0638	0.37 ± 0.06	0.0088 ± 0.0010	27.50 ± 4.45
	B. ovatus	0.50 ± 0.04	0.0163 ± 0.0018	28.50 ± 4.68

[a]Total growth = Max OD_{600} - Min OD_{600}
[b]Specific growth rate = Total growth / Time (Max OD_{600} – Min OD_{600})
[c]Lag time = Time required for culture to increase by OD_{600} 0.1
[d]Only 1 of 3 replicates showed growth; total growth too low to measure lag time

Table S2. Bacterial growth characteristics on glucose and cellooligosaccharides (n = 3).

Carbon source	Strain	Total Growth[a]	Specific Growth Rate(h^{-1})[b]	Lag Time (h)[c]
Glucose	*Bt*	0.80 ± 0.07	0.0300 ± 0.0132	12.33 ± 6.10
	CelUL0638	0.78 ± 0.08	0.0308 ± 0.0152	11.94 ± 5.55
	Bo	0.79 ± 0.09	0.0359 ± 0.0149	13.22 ± 8.44
Cellobiose	*Bt*	0.10[d]	0.0013	N/A
	CelUL0638	0.37 ± 0.06	0.0042 ± 0.0005	47.39 ± 6.44
	Bo	0.83 ± 0.09	0.0201 ± 0.0039	20.50 ± 8.12
Cellotriose	*Bt*	0.14 ± 0.11	0.0018 ± 0.0014	73.17 ± 6.60
	CelUL0638	0.39 ± 0.05	0.0042 ± 0.0007	50.95 ± 11.36
	Bo	0.75 ± 0.10	0.0137 ± 0.0034	39.83 ± 8.21
Cellotetraose	*Bt*	No growth	No growth	No growth
	CelUL0638	No growth	No growth	No growth
	Bo	0.34 ± 0.24	0.0033 ± 0.0023	89.06 ± 12.95
Cellohexaose	*Bt*	No growth	No growth	No growth
	CelUL0638	No growth	No growth	No growth
	Bo	No growth	No growth	No growth

[a]Total growth = Max OD_{600} - Min OD_{600}
[b]Specific growth rate = Total growth / Time (MaxOD_{600} - MinOD_{600})
[c]Lag time = Time required for culture to increase by OD_{600} 0.1
[d]Only 1 of 3 replicates showed growth; total growth too low to measure lag time

Table S3. Bacterial growth characteristics of various strains on glucose and cellobiose (n = 2).

Carbon source	Strain	Total Growth[a]	Specific growth Rate(h^{-1})[b]	Lag time (h)[c]
Glucose	*Bt*	0.74 ± 0.02	0.0185 ± 0.0009	19.71 ± 1.47
	CelUL0638	0.73 ± 0.03	0.0179 ± 0.0010	18.42 ± 1.18
	CelUL0638::P_{BT4615}-*cel94*[d]	0.72 ± 0.01	0.0191 ± 0.0005	16.33 ± 5.07
	Bo	0.84 ± 0.01	0.0280 ± 0.0018	19.79 ± 2.06
CB	*Bt*	No growth	No growth	No growth
	CelUL0638	0.33 ± 0.01	0.0040 ± 0.0002	57.33 ± 2.36
	CelUL0638::P_{BT4615}-*cel94*	0.36 ± 0.02	0.0043 ± 0.0004	66.92 ± 0.00
	Bo	0.79 ± 0.00	0.0162 ± 0.0001	21.88 ± 0.88

[a]Total growth = Max OD_{600} - Min OD_{600}
[b]Specific growth rate = Total growth / Time (MaxOD_{600} - MinOD_{600})
[c]Lag time = Time required for culture to increase by OD_{600} 0.1
[d]Promoter region from *Bt* gene BT4615 replacing cel94 promoter region

Table S4. Bacterial growth characteristics on selected carbon sources for CelUL modifications (n = 3)

Carbon source	Strain	Total growth[a]	Specific trowth rate(h^{-1})[b]	Lag time (h)[c]
Glucose	Bt	0.70 ± 0.04	0.0378 ± 0.0069	5.33 ± 0.60
	CelUL0638	0.71 ± 0.04	0.0366 ± 0.0079	5.78 ± 0.10
	CelUL0638 ΔcelR	0.77 ± 0.02	0.0404 ± 0.0078	5.78 ± 0.35
	CelUL0638 ΔcelR+P6-celR	0.75 ± 0.02	0.0449 ± 0.0130	5.78 ± 0.25
	CelUL0638 P3-celR	0.79 ± 0.05	0.0452 ± 0.0091	5.72 ± 0.42
	CelUL0638 P6-celR	0.82 ± 0.06	0.0435 ± 0.0132	5.39 ± 0.25
	CelUL0638 P3-celT	0.78 ± 0.09	0.0431 ± 0.0144	5.56 ± 0.42
	CelUL0638 P6-celT	0.75 ± 0.05	0.0387 ± 0.0058	5.83 ± 0.00
	CelUL0638 Δcel9	0.83 ± 0.06	0.0460 ± 0.0116	5.50 ± 0.17
	CelUL0638 Δcel9+P6-cel9	0.76 ± 0.08	0.0414 ± 0.0133	5.78 ± 0.10
	Bt P6-cel9	0.81 ± 0.03	0.0492 ± 0.0108	5.83 ± 0.17
	CelUL0638 P3-cel9	0.81 ± 0.08	0.0461 ± 0.0052	6.28 ± 0.35
	CelUL0638 P6-cel9	0.84 ± 0.04	0.0540 ± 0.0077	5.67 ± 0.17
Cellobiose	Bt	No growth	No growth	No growth
	CelUL0638	0.46 ± 0.12	0.0057 ± 0.0017	56.32 ± 8.97
	CelUL0638 ΔcelR	0.42 ± 0.09	0.0052 ± 0.0010	67.76 ± 4.60
	CelUL0638 ΔcelR+P6-celR	0.42 ± 0.08	0.0050 ± 0.0007	46.32 ± 11.45
	CelUL0638 P3-celR	0.42 ± 0.09	0.0049 ± 0.0010	62.65 ± 5.39
	CelUL0638 P6-celR	0.46 ± 0.10	0.0054 ± 0.0013	51.76 ± 11.27
	CelUL0638 P3-celT	0.35 ± 0.17	0.0046 ± 0.0024	61.54 ± 9.56
	CelUL0638 P6-celT	0.67 ± 0.08	0.0097 ± 0.0011	34.39 ± 1.84
	CelUL0638 Δcel9	0.42 ± 0.11	0.0050 ± 0.0014	63.82 ± 5.23
	CelUL0638 Δcel9+P6-cel9	0.37 ± 0.16	0.0044 ± 0.0019	70.60 ± 8.18
	Bt P6-cel9	0.31[d]	0.0036	68.00
	CelUL0638 P3-cel9	0.66 ± 0.15	0.0097 ± 0.0013	43.10 ± 2.41
	CelUL0638 P6-cel9	0.39 ± 0.13	0.0043 ± 0.0014	78.10 ± 5.41
Cellotriose	Bt	0.26 ± 0.03	0.0028 ± 0.0005	45.32 ± 10.07
	CelUL0638	0.48 ± 0.05	0.0067 ± 0.0009	38.21 ± 2.58
	CelUL0638 ΔcelR	-[e]	-	-
	CelUL0638 ΔcelR+P6-celR	-	-	-
	CelUL0638 P3-celR	-	-	-
	CelUL0638 P6-celR	-	-	-
	CelUL0638 P3-celT	-	-	-
	CelUL0638 P6-celT	0.65 ± 0.02	0.0119 ± 0.0009	21.17 ± 2.35
	CelUL0638 Δcel9	0.45 ± 0.05	0.0063 ± 0.0013	47.17 ± 13.20
	CelUL0638 Δcel9+P6-cel9	0.70 ± 0.03	0.0270 ± 0.0041	11.22 ± 3.42
	Bt P6-cel9	0.53 ± 0.06	0.0183 ± 0.0034	12.72 ± 3.53
	CelUL0638 P3-cel9	0.48 ± 0.02	0.0089 ± 0.0011	31.00 ± 1.32
	CelUL0638 P6-cel9	0.57 ± 0.01	0.0198 ± 0.0038	12.94 ± 3.51

[a]Total growth = Max OD_{600} - Min OD_{600}, [b]Specific growth rate = Total growth/Time (MaxOD_{600} - MinOD_{600}), [c]Lag time = Time to increase OD_{600} by 0.1, [d]Only 1 of 3 replicates grew, [e]- = Not measured

Table S5. Strains and plasmids used in this study.

**Strains**			
**Name**	**Genotype**	**Reference**	**Notes**
Bacteroides thetaiomicron			
Bt	tdk- (ΔBT2275)	[1]	Parent "wild-type" strain used in this study; derived from *Bacteroides thetaiotaomicron* VPI-5482/ATCC 29148
*Bt*MLGUL0638	tdk- TR0638::MLGUL	This work	MLGUL from *B. ovatus* ATCC 8483 inserted into the intergenic region between BT0638 and BT0639
CelUL0638	tdk- TR0638::CelUL	This work	CelUL from uncultivated *AC2a* bacterium [2] inserted into the intergenic region between BT0638 and BT0639
CelUL2943	tdk- TR2943::CelUL	This work	CelUL from uncultivated *AC2a* bacterium [2] inserted into the intergenic region between BT2943 and BT2944
CelUL0638 ΔcelR	tdk- TR0638::CelUL ΔcelR	This work	
CelUL0638 ΔcelR+P6-celR	tdk- TR0638::CelUL ΔcelR+P6-celR	This work	P6-celR provided in trans in pNBU2-based vector [3]
CelUL0638 P3-celR	tdk- TR0638::CelUL P3-celR	This work	P3-celR provided in trans in pNBU2-based vector [3]
CelUL0638 P6-celR	tdk- TR0638::CelUL P6-celR	This work	P6-celR provided in trans in pNBU2-based vector [3]
CelUL0638 P3-celT	tdk- TR0638::CelUL P3-celT	This work	P3-celT provided in trans in pNBU2-based vector [3]
CelUL0638 P6-celT	tdk- TR0638::CelUL P6-celT	This work	P6-celT provided in trans in pNBU2-based vector [3]
CelUL0638 Δcel9	tdk- TR0638::CelUL Δcel9	This work	
CelUL0638 Δcel9+P6-cel9	tdk- TR0638::CelUL Δcel9+P6-cel9	This work	P6-cel9 provided in trans in pNBU2-based vector [3]
Bt P6-cel9	*Bt* P6-cel9	This work	P6-cel9 provided in trans in pNBU2-based vector [3]
CelUL0638 P3-cel9	tdk- TR0638::CelUL P3-cel9	This work	P3-cel9 provided in trans in pNBU2-based vector [3]
CelUL0638 P6-cel9	tdk- TR0638::CelUL P6-cel9	This work	P6-cel9 provided in trans in pNBU2-based vector [3]
Others			
Saccharomyces cerevisiae CEN.PK113-5D	MATa ura3-52 MAL2-8c SUC2		For assembly of pICKUP-based plasmids by yeast homologous recombination
Escherichia coli S17-1 *λpir*	TpR SmR recA, thi, pro, hsdR-M+RP4: 2-Tc:Mu: Km Tn7 λpir		For plasmid transformation, replication, and conjugation to *Bacteroides*
Bacteroides ovatus	Wild-type	-	*Bacteroides ovatus* ATCC 8483, type strain; donor for the MLGUL

**Plasmids, name**	**Backbone**	**Reference**	**Notes**
pICKUP	-	This work	Backbone for PUL transfer
pICKUP::TR0638::MLGUL	pICKUP	This work	For transfer of MLGUL to TR0638 targeting region
pICKUP2A	-	This work	Split backbone of pICKUP to reduce background colony formation; part 1 of 2; requires StuI digestion
pICKUP2B	-	This work	Split backbone of pICKUP to reduce background colony formation; part 2 of 2; requires PCR amplification of part

pICKUP2::TR0638::CelUL	pICKUP2	This work	For transfer of CelUL to TR0638 targeting region
pICKUP::TR2943::CelUL	pICKUP	This work	For transfer of CelUL to TR2943 targeting region
pExchange::ΔcelR	pExchange [1]	This work	
pNBU2 P3-celR	pWW3807/pWW3810 [3]	This work	
pNBU2 P6-celR	pWW3807/pWW3810 [3]	This work	
pNBU2 P3-celT	pWW3807/pWW3810 [3]	This work	BsaI restriction sites were removed during Golden Gate assembly
pNBU2 P6-celT	pWW3807/pWW3810 [3]	This work	BsaI restriction sites were removed during Golden Gate assembly
pExchange::Δcel9	pExchange [1]	This work	
pNBU2 P3-cel9	pWW3807/pWW3810 [3]	This work	BsaI restriction sites were removed during Golden Gate assembly
pNBU2 P6-cel9	pWW3807/pWW3810 [3]	This work	BsaI restriction sites were removed during Golden Gate assembly

Table S6. Growth media used in this study

Media	Use	Recipe
Yeast extract peptone dextrose (YPD)	Growth of *S. cerevisiae*	10 g/L yeast extract, 20 g/L peptone, 20 g/L glucose, 20 g/L agar (for plates)
Lysogeny broth (LB)	Growth of *E. coli*	10 g/L tryptone, 10 g/L NaCl, 5 g/L yeast extract, 15 g/L agar (for plates)
Bacteroides minimal medium [4]	Growth assays for *Bacteroides*	100 mM KPO4 (pH 7.2), 15 mM NaCl, 8.5 mM (NH4)2SO4, 4 mM l-cysteine, 1.9 µM hematin, 200 µM l-histidine, 100 µM MgCl2, 1.4 µM FeSO4×7H2O, 50 µM CaCl2, 1 mg/L vitamin K3, 5 µg/L vitamin B12. 5 g/L carbon source (below) as appropriate.
Tryptone yeast extract glucose (TYG)	Growth of *Bacteroides*	Same as *Bacteroides* minimal medium, but with 4 g/L glucose, 10 g/L tryptone and 5 g/L yeast extract added.
Blood agar (BA) plates [5]	Growth and genetic manipulation of *Bacteroides*	37g brain heart infusion (BHI) broth powder and 15g agar (VWR) added to 900 mL of water, autoclaved, cooled until possible to touch, then supplemented with 100 mL of defibrillated horse blood (Håtunalab AB, Solna, Sweden) while stirring. Plates poured to 0.8-1 cm thickness to ensure that they do not dry out during incubation.

Table S7. Primers used in this study. Restriction sites are underlined; Type IIS overhangs are in bold

Name	Sequence
**pICKUP Construction**	
pICKUP T3-1 (amplification of URA3/CEN6/ARS4/kanMX cassette from unpublished vector (C. Kmezik, N. Porter, and J. Larsbrink))	GCGTCTAGACATTTCCCCGAAAAGTGCC
pICKUP T3-2 (amplification of URA3/CEN6/ARS4/kanMX cassette from unpublished vector (C. Kmezik, N. Porter, and J. Larsbrink))	GCGGGATCCGACATGGAGGCCCAGAATAC
**pICKUP2A/B Construction**	
pICKUP Backbone-F SacI	CTCGAGCTCAGGCCTCTAGGTTCCTTTG
pICKUP Backbone-R NheI	GACTAGCTAGCAGGCCTAGCAAGATTTTAAAAGGAAGTATATG
pICKUP4 GFP-F NheI	GATCTGCTAGCGAAAGTGAAACGTGATTTCATGC
pICKUP4 GFP-R SacI	CACGAGCTCTATAAACGCAGAAAGGCCCA
pICKUP4 kanMX-F XbaI	GCGTCTAGAGGTTCTTCTTTCATATACTTCCTT
pICKUP4 URA3_2-R SalI	GTGGTCGACCCTTTTGATGTTAGCAGAATTGTCA
pICKUP4 kanMX-F (amplification of the kanMX/URA3 fragment from pICKUP2B)	GGTTCTTCTTTCATATACTTCCTT
pICKUP-R13 (amplification of the kanMX/URA3 fragment from pICKUP2B)	CCTTTTGATGTTAGCAGAATTGTCA
**pICKUP vectors with PUL insertions**	
MLGUL Frag1-F	CATTCCTGCCATCATTCATGC
MLGUL Frag1-R	GTTGAACGAACGCTTGACATAT
MLGUL Frag2-F	GTCAAGCGATAGTTCAACATATGTC
MLGUL Frag2-R	AGCTTCCGCCATCAGGA
MLGUL Frag3-F	GATGACCTCTGCAGAAGTGAA
MLGUL Frag3-R	GGAGAACGTTTGGCTTATTATGC
CelUL Frag1-F	CTGCAGTTACCGGCCAAGAG
CelUL Frag1-R	CTATGAGAGCGGTATCCGTG
CelUL Frag2-F	GGTTGTGGAAATGCAGAATCC
CelUL Frag2-R	GATTGATCATGCTACAGGACGG
CelUL Frag3-F	GCACTGTTTAAGTGAGTGTCTC
CelUL Frag3-R	GGATCCTCAGAACTGCTCG
P1 TR BT0638-F2000	TGAAGCAGGCGGCAGAAGAAGTAACAAAGGAACCTAGAGGCGAGTAGGCCTCCGAAT
P1 TR BT0638-F750	TGAAGCAGGCGGCAGAAGAAGTAACAAAGGAACCTAGAGGGAACGATCATCATTCCTGTTC

P2 TR BT0638-R MLGUL	AAATTCTCCGGGGAACCAGGCATGAATGATGGCAGGAATGAGGAATCACATGATAAACGATTTCA
P2 TR BT0638-R CelUL	CCTCTCCGCCGGCCTTGACGCTCTTGGCCGGTAACTGCAGAGGAATCACATGATAAACGATTTCA
P3 TR BT0638-F MLGUL	CGTAAGTTTTGGCCAATGCATAATAAGCCAAACGTTCTCCTTTTTACCATTCATAAGAAATACAGA
P3 TR BT0638-F CelUL	TTGCACGCCATCGGAGTTCCGCGAGCAGTTCTGAGGATCCTTTTTACCATTCATAAGAAATACAGA
P4 TR BT0638-R750	TAGGGAGCCCTTGCATGACAATTCTGCTAACATCAAAAGGCACGATAAATGTGCTTAATACAC
P4 TR BT0638-R2000	TAGGGAGCCCTTGCATGACAATTCTGCTAACATCAAAAGGGTTATCCATCAGCATGGCA
**qPCR primers**	
BACOVA_2742 qPCR-F	TCCGTGGAAATGGGATTGGA
BACOVA_2742 qPCR-R	CGTGTGCTAAGGGCAATCTC
AC2aCelC qPCR-F	AAACATTGTGACGGGCGTTG
AC2aCelC qPCR-R	TCGTCACCGATATTCGCCTG
AC2aGH94 qPCR-F	TGGCGAGCAATCGACCTATC
AC2aGH94 qPCR-R	TGATTCTCGATCCATGCGGG
AC2aCelE qPCR-F	TGCTTCGTCGGTTACGTTCA
AC2aCelE qPCR-R	GTCCTGGTTGAGCATCGACA
**CelUL deletion constructs**	
celR left F	GCGGGTCTCCGTTCCAATAAGGTATTCGTAAAGAAGACAGAAC
celR left R	GCGGGTCTCGATGTGAGGATCCTTTTTACCATTCATAAG
celR right F	GCGGGTCTCCACATGGCTGATAGCAAATGGCC
celR right R	GCGGGTCTCGCACTCGTGACGGCACTCATAGTTG
celT left F	GCGGGTCTCCGTTCGACCGGTGCAATGTCCG
celT left R	GCGGGTCTCCTCGCGGCATCACCCAGAGCATAA
celT right F	GCGGGTCTCGGCGATCATGGGCTGGC
celT right R	GCGGGTCTCGCACTCTGTTGAGATGCAGAATGCTTG
cel9 left F	GCGGGTCTCCGTTCGCACATCCTCAACCGGTC
cel9 left R	GCGGGTCTCGTCGGCACAAGCACTTAGAATGC
cel9 right F	GCGGGTCTCCCCGACGCTCTTGGCCGG
cel9 right R	GCGGGTCTCGCACTGCACTCTCACGGATTCCTTC
**CelUL overexpression constructs**	
celR part-F	ATCGTCTCAACTAGGTCTCGAATGAAAGAATCATTCACCGAAATTACC
celR part-R	TACGTCTCCAAGCGGTCTCGGGATTCAGAACTGCTCGCGGA
celT part 1-F	ATCGTCTCAACTAGGTCTCGAATGGCTCCGTTTCGCG

celT part 1-R	TACGTCTCCTCCGACGCCAATAGACGTG
celT part 2-F	ATCGTCTCACGGACTCTACTCCCCGCTGATCTG
celT part 2-R	TACGTCTCCAAGCGGTCTCGGGATCGTTATTTATGCAAAGGGAATTTATATGC
cel9 part 1-F	ATCGTCTCAACTAGGTCTCGAATGAAAAAGATATTTTGCATTCTAAGTGC
cel9 part 1-R	TACGTCTCCTGTCTCGGCATCCTGGC
cel9 part 2-F	ATCGTCTCAGACAGATGGTGTGATCTACCCGAAG
cel9 part 2-R	TACGTCTCCAAGCGGTCTCGGGATGTTACCGGCCAAGAGCG

Supplementary references

1. Koropatkin NM, Martens EC, Gordon JI, Smith TJ. Starch catabolism by a prominent human gut symbiont is directed by the recognition of amylose helices. Structure. 2008;16(7):1105-15; doi: 10.1016/j.str.2008.03.017.
2. Hess M, Sczyrba A, Egan R, Kim TW, Chokhawala H, Schroth G, et al. Metagenomic discovery of biomass-degrading genes and genomes from cow rumen. Science. 2011;331(6016):463-7; doi: 10.1126/science.1200387.
3. Whitaker WR, Shepherd ES, Sonnenburg JL. Tunable expression tools enable single-cell strain distinction in the gut microbiome. Cell. 2017;169(3):538-46.e12; doi: 10.1016/j.cell.2017.03.041.
4. Martens EC, Chiang HC, Gordon JI. Mucosal glycan foraging enhances fitness and transmission of a saccharolytic human gut bacterial symbiont. Cell Host Microbe. 2008;4(5):447-57; doi: 10.1016/j.chom.2008.09.007.
5. Martens EC, Lowe EC, Chiang H, Pudlo NA, Wu M, McNulty NP, et al. Recognition and degradation of plant cell wall polysaccharides by two human gut symbionts. PLoS Biol. 2011;9(12):e1001221; doi: 10.1371/journal.pbio.1001221.
